# Peggy Seriès: Bayesian on a bike

**DOI:** 10.1192/bjb.2021.91

**Published:** 2021-12

**Authors:** Claire McKenna

**Figure:**
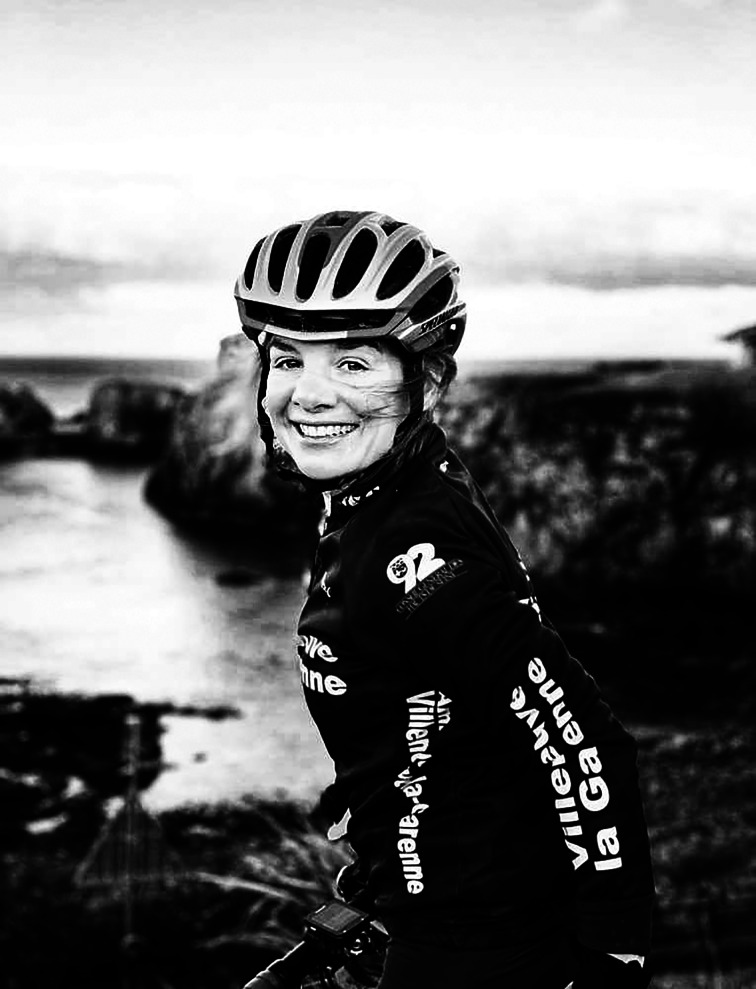

Born near Bordeaux in 1974, Dr Peggy Seriès is a senior lecturer and principal investigator in the computational psychiatry laboratory at the Institute for Adaptive and Neural Computation (ANC), University of Edinburgh. Her career to date has included spells in prestigious computational neuroscience labs in France, New York and University College London.

Her move into computational psychiatry was motivated, she says, partly by her own experience of anxiety, but also by witnessing the suffering due to mental illness of her students and the death by suicide of two of them.

Essentially, the Bayesian brain hypothesis suggests that brains use probability calculations to make *predictions* about what we experience, based on sensory inputs and our prior experiences. Seriès's research work focuses on using mathematical and computer models to understand how our expectations and prior beliefs about the world modulate our perception. In particular she is interested in differences in prior beliefs and learning in disorders such as schizophrenia, autism, depression and anxiety.

Seriès edited and contributed to the first accessible textbook on the emerging field of computational psychiatry.^1^ For any clinicians feeling jaded, read it for an overview of computational approaches in your psychiatric field of interest and to get excited about psychiatry all over again.

Seriès only started cycling competitively at the age of 39. Since then she has won multiple track cycling championships nationally and internationally, completed three half ironmans and is in training for a full ironman. In 2013 she cycled the full route of the Tour de France, considered one of the most gruelling endurance competitions in the world.

This interview took place via Zoom in June 2021 and has been edited for length and clarity.

**I want to hear more about your cycling achievements! Is there a connection between your love of cycling and your interest in maths and neuroscience?**

Thanks for asking! I'm actually very proud of my cycling achievements, mostly because I started competing so late in life and it was never something I thought I could do. Racing bikes has been such a blast!

I'm not sure there is a clear connection, though maybe the pleasure I get from both is the pleasure of feeling like an explorer, which is what I wanted to be when I grew up. On the bike, what I like best is putting my panniers on, carrying a tent and going for an adventure.

**You diverged from an undergraduate degree in maths and physics into computational neuroscience – was that always your plan?**

No, it's been a tortuous path, via electronic engineering and artificial intelligence, but I'm very happy about where I landed.

I think the deciding factor was that my father suffered an encephalitis when I was 19. For a while he had short-term memory loss, olfactory hallucinations and prosopagnosia. For me, it was a shocking awareness of how delicate and complex the brain is and how a lot of our self-identity is related to our memories: who are we if/when we lose our memories? Initially, memory was what I wanted to study.

**Did anyone else inspire you?**

My mother was a neurologist but has always been fascinated by psychiatry, so she'd have to be on the list. Otherwise, in the people I've worked with, I'd have to say Peter Dayan for his pioneering and very inspiring work in computational psychiatry and Eero Simoncelli, who has been a role model as a researcher and mentor.

**What gets you out of bed in the morning?**

Mostly porridge! After breakfast though, I think I am fascinated by understanding how one's experience of life is shaped. How free are we about how we feel, how happy we are? This is something I am working on in my private life, having more control over my own experience, and I see it related to my work as well, both my work on Bayesian perception and on mental illness: how much are we shaped by our previous beliefs? Where do those beliefs come from? How can we change them?

**What keeps you awake at night?**

That the causes of mental illness may lie more in how our society pressures us and lets the vulnerable down than in how our brain might become ‘chemically unbalanced’. That all the neuroscience understanding in the world can't compensate for a society that makes people feel stressed and miserable.

**What do you love most about what you do?**

I'd have to say interacting with students, seeing my PhD students develop. In my teaching, having an honest discussion about mental illness, when they are at an age when it starts to be relevant – an age at which I certainly would have liked to be more informed about it.

**Computational neuroscience still seems to be a field dominated by men – have you had any difficulties as a woman in this field?**

This year a woman has been recruited [to the ANC] but until now I have been the only woman in my Institute out of 20 people. I think it's at the current stage of my career that I see how male-dominated things are in academia and when I feel that progression is harder. Or maybe I was more naive before. There's a perception that women don't put themselves forward as much as men do, and I think this was definitely true of me, and it came at a cost.

I also think it is undeniable that it is hard for women to succeed in academia while at the same time managing to settle down in one place and with one person to build a family.

You asked me about the connection between my work in neuroscience and my passion for cycling. I think the cycling gave me a lot of confidence. I am more confident now in this world of men knowing that on a bike I can actually beat them! It's as if I got on their kind of field and I am actually competitive.

**Do you think things are any different now for young female academics than they were for you?**

Yes, I think things are different now. Women were very clearly treated differently from men when I was young. It was so ingrained in our society that we (women) were not even aware of it.

A very long time ago during my studies, I was supposed to be supervised for a small research project by a supervisor who, in the first meeting with me, invited me for a drink and asked if I had a boyfriend. On our second meeting, he was sitting topless in his office, feet on the table and when I came through the door, commented that I was short. I never went back but also never thought to tell anyone about this – I just did my project alone.

It is now covert and more subtle and probably takes different forms – I'd like to think that behaviours like this don't happen anymore, but as in the rest of society, I think in academia women still feel they are playing a game where the rules have been written mostly by, and for, men.

**Thank you for sharing that. I was reminded of the ♯BalanceTonPorc movement in France, the equivalent of the ♯MeToo movement. Did you ever consider denouncing your aggressor?**

No. You know back then I just felt happy that I could work by myself and I didn't realise what it was. But now I think about it as a supervisor myself. If I imagine behaving like this with a student of mine, it's very difficult to understand.

**You've clearly done a lot to recruit, do research with and nurture PhD students. You've also written about the importance of ‘scientific humility’ and making science more accessible. Perhaps this humility doesn't serve women well in a field dominated by men. You have though used your skills of collaboration to pull together international leaders in the field of computational psychiatry for your new book. Why did you think there was a need for a primer?**

The field has been growing steadily in the past few years, but it is not often taught formally yet. I wanted to offer a book that could be accessible to a broad audience. I had my students in mind but it is hopefully also accessible to students coming from psychology or medicine. The book is not perfect, but I think it's a start and hopefully it will help to consolidate the field and lead to better things.

I came from a background where people often like to explain things in a very complicated way with very complicated vocabulary. I have been inspired by researchers like Eero Simoncelli, who instead would say ‘it needs to be clear to your grandmother’. That was the contrary of arrogance in how you present your work – the need to aim for accessibility. For me, it was very inspiring.

**Why did you use that Einstein quotation at the start of the chapter you wrote:^1^ ‘One thing I have learned in a long life is that all our science, measured against reality is primitive and childlike – and yet it is the most precious thing we have’?**

What I meant is that the models we have at the moment are most certainly oversimplified and naive, but we have to start somewhere and from there we can grow and improve.

**I want you to give me a bit of an ‘idiot's guide’ to computational psychiatry! How are computational theories of the mind linked to computers – is it anything to do with computer (hardware/software) metaphors of the mind?**

In some sense – the computational theory of mind views the mind as an information processing system, and perception and cognition as a form of computation that is realised by neural activity in the brain. The software would be cognition and the hardware, the neurons.

The work we do operates at both levels: how can we describe cognitive processes and how are those cognitive processes realised by the neural substrate?

**Language is a limited and imprecise way to describe how our brain and mind works. Is computation a tool to extend our own mental capacity and escape the imprecision of words?**

Yes, verbal theories can only make general and somewhat vague predictions. Mathematical descriptions offer a way to formalise theories precisely and quantitatively so they can be tested, compared with data and extended.

**Computational psychiatry has been called a ‘Rosetta stone’ linking levels of explanation in mental illness – do you agree?**

I don't think it will lead to an overnight understanding of mental illness. But I think computational neuroscience tools are very good at exploring and providing links between different levels of description, for example linking the description of individual neurons and the dynamics of networks of neurons, then linking networks of neurons and behaviour etc. In that sense they can provide links between neurobiological elements and descriptions related to symptoms.

**Why is Bayes’ rule important in understanding the mind?**

Bayes’ theorem tells us how to optimally calculate the probability of an event based on new information that is, or may be, related to that event, as well as prior information.

It is important in understanding how the mind works because the mind has to do something like that: figure out at each moment in time what is in the environment and what is the best action to take. It has to combine uncertain bits of information and try to make sense of that information in view of previous knowledge. Bayesian inference can thus offer a benchmark of how the brain (I notice you say the mind and I say the brain!) should do that if it were functioning optimally. It is often found that this benchmark comes close to what the brain does in practice.

**What is the difference between Bayesian inference and predictive processing?**

Predictive processing, or predictive coding, is a theory of brain function in which the brain is constantly generating and updating an internal model of the environment. The model is used to generate predictions of sensory input that are compared with actual sensory input. This comparison results in prediction errors that are then used to update and revise the mental model.

Bayesian inference is a method of statistical inference in which Bayes’ theorem is used to update the probability for a hypothesis as more evidence or information becomes available.

Although the neuroscience literature often confuses the two, as both theories are related to building an internal model of the environment to generate predictions, they are distinct. Predictive processing can implement Bayesian inference, but it does not necessarily do so, nor would it be the only way to implement Bayesian inference.

**What is machine learning and how is this used in computational psychiatry?**

Machine learning is a branch of artificial intelligence based on the idea that systems can learn from data, identify patterns in the data and make decisions with minimal human intervention.

We distinguish two types of computational psychiatry: theory-driven and data-driven.

The first kind uses mostly models coming from computational neuroscience, that formalise psychological or neural hypotheses about how the brain learns or makes decisions and produces behaviour (such as reinforcement learning models and Bayesian models). This is the type I am mostly involved in.

The second kind uses machine learning to try to ‘blindly’ detect patterns in psychiatric data or do prediction or classification (without making assumptions about the underlying mechanisms).

Both types of work are complementary and can also be combined.

**What translational benefit has computational psychiatry had?**

There are already indications that machine learning can help predict trajectories of mental illness or predict response to treatment. There is for example a seminal study by Chekroud et al,^2^ showing that it is possible to predict (somewhat significantly above chance) response to treatment (citalopram) for people suffering from depression. But overall, the field is really in its infancy.

**You pointed out that you tend to talk about ‘the brain’ and I tend to say ‘the mind’. Is computational psychiatry more concerned with the computational representation of cognition, as opposed to the qualia of consciousness?**

Yes, we computational neuroscientists commonly like to avoid talking about qualia or even consciousness, we often feel uncomfortable around those concepts and like to leave those to philosophers of the mind! I think it's a shame that it's not a literature we often read and we are not enough exposed to it. Philosophers of mind read us, but we don't really read them.

I think, though, it will be very important to address the notion of suffering and mental pain. At the end of the day it is not really the content of the experience that makes a person feel ‘ill’ – some people have hallucinations they are perfectly comfortable with – but that feeling of suffering, that something is ‘very wrong’ or overwhelming, that is perhaps difficult to measure or model quantitatively.

**What projects are you currently working on?**

One of my main projects is about clarifying the differences between Bayesian theories in relation to autism and schizophrenia: the theories are quite similar at the moment for the two disorders, while the symptoms are very different.

**As far as I understand it, the similarities are related to the idea that in both autism and schizophrenia there are ‘weak priors’, so that the influence of prior expectations on perception is weaker?**

Yes, exactly. The ideas are very similar, that you'd have a weak influence of perceptual priors in both autism and schizophrenia, but perhaps at a more cognitive level you would have stronger (possibly maladaptive and overwhelming) priors in schizophrenia. But in reality the pathologies have very rarely been compared using computational methods.

I'm really interested in actually testing these theories. And what's interesting is that in our work we found differences [between autism, schizophrenia and controls], but they are very subtle. And I find that quite fascinating as well.

In reality, even between schizophrenia, autism, depression and anxiety, it's very hard to find behavioural tasks that lead to very robust differences that we can actually quantify and model. So at the moment, there is some indication that these theories are somewhat promising, but there's also data coming in that are against those theories. It's quite hard to know how much progress we have really made, I think, with these Bayesian theories and part of my work tries to assess that.

**The Bayesian framework for understanding the mind seems intuitively appealing, and can be retrofitted to lots of psychological theories. However, it is often applied very liberally, in a speculative kind of way that seems unfalsifiable. The more I read about predictive processing, the more I can apply it to just about anything! Is there a risk of the Bayesian bandwagon running away with us?**

As a framework, the Bayesian approach is indeed unfalsifiable; there will always be a Bayesian model that can fit the data. However, individual models are falsifiable. I think the trick is not to use Bayesian or predictive ideas in the vague sense, but to formalise these ideas as much as possible in the form of mathematical models and simulations and compare a family of different individual Bayesian models and other types of model. Only then can we test quantitatively our hypotheses, confront the data and really evaluate whether/which Bayesian descriptions really correspond to the data.

**We may be able to understand the neural mechanisms behind the distress related to psychiatric disorders, but do computational approaches to understanding mental illness tell us at what level we should intervene to relieve this distress? It may still be best to intervene at the level of people's socioeconomic circumstances or preventing their trauma or challenging their negative thought patterns.**

I agree, but I don't think these levels of explanation are incompatible. A computational approach (such as machine learning) can be informative in identifying what environmental factors are critical in triggering mental illness, for example.

Work looking at reinforcement learning and Bayesian inference can also hopefully ultimately inform learning-based psychotherapies, as researchers like Michael Moutoussis^3^ have shown.

**If computational psychiatry were the Tour de France, which stage are we currently at? What would represent the Champs-Élysées in computational psychiatry for you?**

We're probably at the Prologue [the time trial which starts the Tour] even if it feels like the Alps! The Champs-Elysées, for me, would be to better understand the root causes of mental illness, either at a biological or environmental level, and inform new therapies, in particular psychotherapies.
